# (2*Z*)-1-(5-Hy­droxy-3-methyl-1-phenyl-1*H*-pyrazol-4-yl)-3-(4-meth­oxy­anilino)but-2-en-1-one

**DOI:** 10.1107/S1600536811032491

**Published:** 2011-08-17

**Authors:** Abdullah M. Asiri, Abdulrahman O. Al-Youbi, Hassan M. Faidallah, Seik Weng Ng, Edward R. T. Tiekink

**Affiliations:** a Chemistry Department, Faculty of Science, King Abdulaziz University, PO Box 80203, Jeddah, Saudi Arabia; b The Center of Excellence for Advanced Materials Research, King Abdulaziz University, Jeddah, PO Box 80203, Saudi Arabia; c Department of Chemistry, University of Malaya, 50603 Kuala Lumpur, Malaysia

## Abstract

The central residue in the title compound, C_21_H_21_N_3_O_3_, is close to planar (r.m.s. deviation = 0.0753 Å for all non-H atoms from OH to NH inclusive): the hy­droxy, amino and carbonyl groups all lie to the same side of the mol­ecule (the conformation about the ethene bond is *Z*), facilitating the formation of intra­molecular O—H⋯O and N—H⋯O hydrogen bonds that close *S*(6) rings. However, overall the mol­ecule is twisted as the terminal aromatic rings are not coplanar with the central plane [dihedral angles = 20.55 (5) and 80.90 (4)° for the N-bound phenyl ring and the meth­oxy­benzene ring, respectively]. The dihedral angle between the rings is 82.14 (7)°. Supra­molecular layers in the *ac* plane mediated by C—H⋯π inter­actions are found in the crystal.

## Related literature

For background to the synthesis, see: Gelin *et al.* (1983 ▶); Bendaas *et al.* (1999 ▶). For the structure of the 4-chloro derivative, see: Asiri *et al.* (2011 ▶).
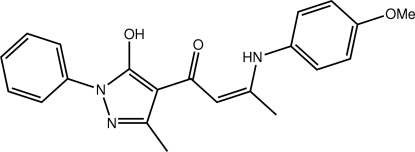


## Experimental

### 

#### Crystal data

C_21_H_21_N_3_O_3_
*M* *_r_* = 363.41
Monoclinic, 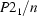

*a* = 9.5717 (3) Å
*b* = 16.9516 (6) Å
*c* = 11.3143 (4) Å
β = 104.946 (4)°
*V* = 1773.70 (10) Å^3^
*Z* = 4
Mo *K*α radiation
μ = 0.09 mm^−1^
*T* = 100 K
0.30 × 0.25 × 0.20 mm

#### Data collection

Agilent SuperNova Dual diffractometer with an Atlas detector
Absorption correction: multi-scan (*CrysAlis PRO*; Agilent, 2010 ▶) *T*_min_ = 0.837, *T*_max_ = 1.000
8486 measured reflections
3939 independent reflections
3145 reflections with *I* > 2σ(*I*)
*R*_int_ = 0.024

#### Refinement

*R*[*F* ^2^ > 2σ(*F* ^2^)] = 0.042
*wR*(*F* ^2^) = 0.104
*S* = 1.05
3939 reflections
255 parameters
2 restraints
H atoms treated by a mixture of independent and constrained refinement
Δρ_max_ = 0.27 e Å^−3^
Δρ_min_ = −0.23 e Å^−3^

### 

Data collection: *CrysAlis PRO* (Agilent, 2010 ▶); cell refinement: *CrysAlis PRO*; data reduction: *CrysAlis PRO*; program(s) used to solve structure: *SHELXS97* (Sheldrick, 2008 ▶); program(s) used to refine structure: *SHELXL97* (Sheldrick, 2008 ▶); molecular graphics: *ORTEP-3* (Farrugia, 1997 ▶) and *DIAMOND* (Brandenburg, 2006 ▶); software used to prepare material for publication: *publCIF* (Westrip, 2010 ▶).

## Supplementary Material

Crystal structure: contains datablock(s) global, I. DOI: 10.1107/S1600536811032491/hb6355sup1.cif

Structure factors: contains datablock(s) I. DOI: 10.1107/S1600536811032491/hb6355Isup2.hkl

Supplementary material file. DOI: 10.1107/S1600536811032491/hb6355Isup3.cml

Additional supplementary materials:  crystallographic information; 3D view; checkCIF report

## Figures and Tables

**Table 1:** Hydrogen-bond geometry (Å, °) *Cg*1 and *Cg*2 are the centroids of the N1,N2,C1–C3 and C15–C20 rings, respectively.

*D*—H⋯*A*	*D*—H	H⋯*A*	*D*⋯*A*	*D*—H⋯*A*
O1—H1⋯O2	0.86 (1)	1.68 (1)	2.4963 (15)	156 (2)
N3—H3⋯O2	0.89 (1)	1.92 (1)	2.6447 (16)	138 (2)
C14—H14b⋯*Cg*1^i^	0.98	2.88	3.5542 (18)	127
C21—H21c⋯*Cg*2^ii^	0.98	2.76	3.5195 (17)	134

Symmetry codes: (i) 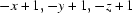; (ii) 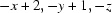.

**Fig. 1.**
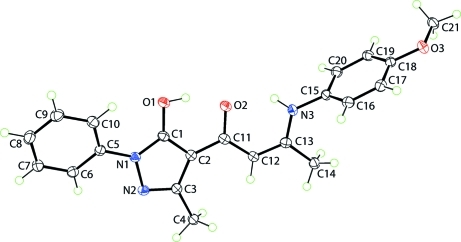
The molecular structure of (I) showing displacement ellipsoids at the 50% probability level.

**Fig. 2.**
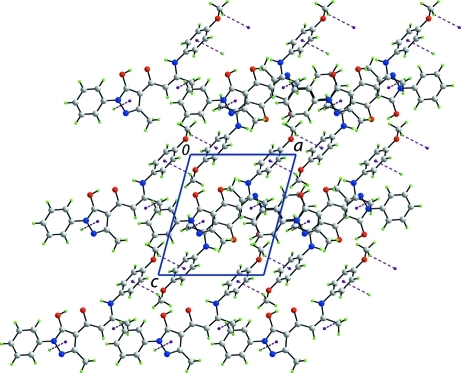
A view of a supramolecular layer in (I) mediated by C—H···π interactions, shown as purple dashed lines

**Fig. 3.**
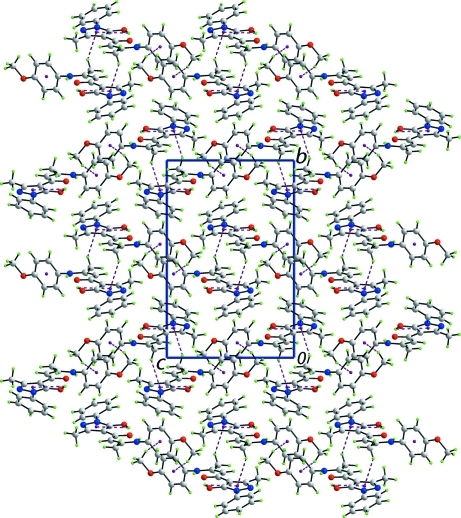
Stacking of supramolecular layers along the b axis in (I). The C—H···π interactions are shown as purple dashed lines.

## supplementary crystallographic information

### Comment

In connection with a recent structural study (Asiri *et al.*, 2011), the title compound (I) was prepared as a part of on-going investigations of reactions between pyrazoles and aniline derivatives (Gelin *et al.*, 1983; Bendaas *et al.*, 1999).

The molecular structure of (I), Fig. 1, resembles closely that of the 4-chloroanilino derivative (Asiri *et al.*, 2011) and features a *Z* configuration about the C12—C13 [1.381 (2) Å] bond. The hydroxy and amino groups are *syn* to the central carbonyl group and each forms a hydrogen bond to close a *S*(6) ring (Table 1). A direct consequence of this is that the central residue is planar; the values of the C1—C2—C11—O2, C2—C11—C12—C13 and C11—C12—C13—N3 torsion angles are -3.6 (2), -171.74 (15) and -1.7 (2) °, respectively. The benzene and 4-methoxybenzene rings are each twisted out of the central plane as seen in the values of the C1—N1—C5—C6 and C13—N3—C15—C16 torsion angles of 159.97 (15) and -74.5 (2) °, respectively.

The most prominent feature of the crystal packing is the formation of supramolecular layers in the *ac* plane and mediated by C—H···π interactions, Fig. 2. and Table 1. Layers stack along the *b* axis as shown in Fig. 3.

### Experimental

A solution of 4-acetoacetyl-5-hydroxy-3-methyl-1-*p*-sulfamylphenypyrazole (1.7 g, 0.005 mole) and 4-methoxyaniline (0.63 g, 0.005 mole) in ethanol (25 ml) was refluxed for 2 h. The precipitate, obtained from the hot solution, was collected, washed with methanol and recrystallized from ethanol-benzene as yellow blocks; *M*.pt: 507–507 K.

### Refinement

Carbon-bound H-atoms were placed in calculated positions [C—H 0.95 to 0.98 Å, *U*_iso_(H) 1.2 to 1.5*U*_eq_(C)] and were included in the refinement in the riding model approximation. The hydroxyl- and amino- H-atoms were located in a difference Fourier map, and subsequently refined freely.

### Crystal data

**Table:**

C_21_H_21_N_3_O_3_	*F*(000) = 768
*M**_r_* = 363.41	*D*_x_ = 1.361 Mg m^−^^3^
Monoclinic, *P*2_1_/*n*	Mo *K*α radiation, λ = 0.71073 Å
Hall symbol: -P 2yn	Cell parameters from 3917 reflections
*a* = 9.5717 (3) Å	θ = 2.4–29.3°
*b* = 16.9516 (6) Å	µ = 0.09 mm^−^^1^
*c* = 11.3143 (4) Å	*T* = 100 K
β = 104.946 (4)°	Block, yellow
*V* = 1773.70 (10) Å^3^	0.30 × 0.25 × 0.20 mm
*Z* = 4	

### Data collection

**Table:**

Agilent SuperNova Dual diffractometer with an Atlas detector	3939 independent reflections
Radiation source: SuperNova (Mo) X-ray Source	3145 reflections with *I* > 2σ(*I*)
mirror	*R*_int_ = 0.024
Detector resolution: 10.4041 pixels mm^-1^	θ_max_ = 27.5°, θ_min_ = 2.4°
ω scans	*h* = −12→9
Absorption correction: multi-scan (*CrysAlis PRO*; Agilent, 2010)	*k* = −17→21
*T*_min_ = 0.837, *T*_max_ = 1.000	*l* = −14→14
8486 measured reflections	

### Refinement

**Table:**

Refinement on *F*^2^	Primary atom site location: structure-invariant direct methods
Least-squares matrix: full	Secondary atom site location: difference Fourier map
*R*[*F*^2^ > 2σ(*F*^2^)] = 0.042	Hydrogen site location: inferred from neighbouring sites
*wR*(*F*^2^) = 0.104	H atoms treated by a mixture of independent and constrained refinement
*S* = 1.05	*w* = 1/[σ^2^(*F*_o_^2^) + (0.0364*P*)^2^ + 0.8331*P*] where *P* = (*F*_o_^2^ + 2*F*_c_^2^)/3
3939 reflections	(Δ/σ)_max_ < 0.001
255 parameters	Δρ_max_ = 0.27 e Å^−^^3^
2 restraints	Δρ_min_ = −0.23 e Å^−^^3^

### Special details

**Table:**

Geometry. All e.s.d.'s (except the e.s.d. in the dihedral angle between two l.s. planes) are estimated using the full covariance matrix. The cell e.s.d.'s are taken into account individually in the estimation of e.s.d.'s in distances, angles and torsion angles; correlations between e.s.d.'s in cell parameters are only used when they are defined by crystal symmetry. An approximate (isotropic) treatment of cell e.s.d.'s is used for estimating e.s.d.'s involving l.s. planes.
Refinement. Refinement of *F*^2^ against ALL reflections. The weighted *R*-factor *wR* and goodness of fit *S* are based on *F*^2^, conventional *R*-factors *R* are based on *F*, with *F* set to zero for negative *F*^2^. The threshold expression of *F*^2^ > σ(*F*^2^) is used only for calculating *R*-factors(gt) *etc*. and is not relevant to the choice of reflections for refinement. *R*-factors based on *F*^2^ are statistically about twice as large as those based on *F*, and *R*- factors based on ALL data will be even larger.

### Fractional atomic coordinates and isotropic or equivalent isotropic displacement parameters (Å^2^)

**Table:**

	*x*	*y*	*z*	*U*_iso_*/*U*_eq_	
O1	0.14980 (12)	0.66071 (7)	0.33592 (9)	0.0222 (3)	
O2	0.37382 (11)	0.60631 (7)	0.29255 (9)	0.0213 (3)	
O3	0.91460 (11)	0.58562 (7)	−0.12793 (9)	0.0219 (3)	
N1	0.17619 (13)	0.67412 (8)	0.54868 (11)	0.0166 (3)	
N2	0.28248 (13)	0.65978 (8)	0.65708 (11)	0.0175 (3)	
N3	0.61937 (14)	0.57378 (8)	0.23667 (11)	0.0195 (3)	
C1	0.22616 (16)	0.65491 (9)	0.45138 (13)	0.0170 (3)	
C2	0.36749 (16)	0.62804 (9)	0.49321 (13)	0.0169 (3)	
C3	0.39577 (15)	0.63279 (9)	0.62323 (13)	0.0162 (3)	
C4	0.52961 (16)	0.61127 (10)	0.71833 (13)	0.0196 (3)	
H4A	0.5134	0.6184	0.7997	0.029*	
H4B	0.6094	0.6452	0.7101	0.029*	
H4C	0.5540	0.5560	0.7078	0.029*	
C5	0.04095 (15)	0.70640 (9)	0.55398 (13)	0.0166 (3)	
C6	−0.00329 (16)	0.69838 (9)	0.66119 (13)	0.0187 (3)	
H6	0.0562	0.6716	0.7296	0.022*	
C7	−0.13461 (16)	0.72972 (10)	0.66734 (14)	0.0217 (3)	
H7	−0.1651	0.7245	0.7405	0.026*	
C8	−0.22213 (17)	0.76870 (10)	0.56770 (15)	0.0247 (4)	
H8	−0.3126	0.7898	0.5722	0.030*	
C9	−0.17707 (17)	0.77671 (10)	0.46184 (14)	0.0235 (4)	
H9	−0.2367	0.8036	0.3936	0.028*	
C10	−0.04527 (17)	0.74579 (10)	0.45410 (14)	0.0205 (3)	
H10	−0.0146	0.7516	0.3811	0.025*	
C11	0.44652 (16)	0.60431 (9)	0.40619 (13)	0.0176 (3)	
C12	0.59321 (16)	0.58162 (9)	0.44007 (13)	0.0175 (3)	
H12	0.6385	0.5752	0.5247	0.021*	
C13	0.67514 (16)	0.56811 (9)	0.35792 (13)	0.0176 (3)	
C14	0.83048 (16)	0.54384 (10)	0.40247 (14)	0.0211 (3)	
H14A	0.8907	0.5790	0.3677	0.032*	
H14B	0.8417	0.4895	0.3769	0.032*	
H14C	0.8606	0.5471	0.4919	0.032*	
C15	0.70003 (15)	0.57341 (10)	0.14592 (13)	0.0179 (3)	
C16	0.77859 (16)	0.63978 (10)	0.13037 (13)	0.0193 (3)	
H16	0.7840	0.6838	0.1834	0.023*	
C17	0.84893 (16)	0.64190 (10)	0.03781 (13)	0.0190 (3)	
H17	0.9022	0.6874	0.0271	0.023*	
C18	0.84160 (15)	0.57738 (9)	−0.03966 (13)	0.0172 (3)	
C19	0.76391 (16)	0.51053 (10)	−0.02410 (13)	0.0193 (3)	
H19	0.7589	0.4663	−0.0766	0.023*	
C20	0.69349 (16)	0.50912 (10)	0.06938 (13)	0.0196 (3)	
H20	0.6405	0.4636	0.0807	0.023*	
C21	0.90919 (17)	0.52107 (10)	−0.21023 (13)	0.0223 (3)	
H21A	0.9647	0.5343	−0.2692	0.033*	
H21B	0.8084	0.5106	−0.2540	0.033*	
H21C	0.9507	0.4740	−0.1640	0.033*	
H1	0.214 (2)	0.6449 (15)	0.300 (2)	0.067 (8)*	
H3	0.5266 (11)	0.5872 (11)	0.2149 (16)	0.030 (5)*	

### Atomic displacement parameters (Å^2^)

**Table:**

	*U*^11^	*U*^22^	*U*^33^	*U*^12^	*U*^13^	*U*^23^
O1	0.0225 (6)	0.0292 (7)	0.0142 (5)	0.0037 (5)	0.0039 (4)	−0.0013 (5)
O2	0.0214 (5)	0.0273 (6)	0.0154 (5)	0.0025 (5)	0.0049 (4)	−0.0012 (4)
O3	0.0259 (6)	0.0232 (6)	0.0198 (5)	−0.0003 (5)	0.0119 (5)	0.0004 (4)
N1	0.0172 (6)	0.0178 (7)	0.0141 (6)	0.0020 (5)	0.0027 (5)	0.0006 (5)
N2	0.0187 (6)	0.0179 (7)	0.0149 (6)	0.0010 (5)	0.0024 (5)	0.0007 (5)
N3	0.0178 (6)	0.0250 (8)	0.0171 (6)	0.0018 (6)	0.0070 (5)	−0.0006 (5)
C1	0.0207 (7)	0.0155 (8)	0.0150 (7)	−0.0020 (6)	0.0048 (6)	−0.0008 (6)
C2	0.0185 (7)	0.0158 (8)	0.0166 (7)	−0.0001 (6)	0.0050 (6)	0.0000 (6)
C3	0.0181 (7)	0.0138 (7)	0.0172 (7)	−0.0021 (6)	0.0055 (6)	−0.0004 (6)
C4	0.0204 (7)	0.0215 (8)	0.0172 (7)	0.0024 (6)	0.0053 (6)	−0.0002 (6)
C5	0.0159 (7)	0.0145 (8)	0.0193 (7)	−0.0009 (6)	0.0042 (6)	−0.0029 (6)
C6	0.0183 (7)	0.0189 (8)	0.0184 (7)	−0.0013 (6)	0.0036 (6)	−0.0002 (6)
C7	0.0201 (7)	0.0254 (9)	0.0208 (7)	−0.0020 (7)	0.0075 (6)	−0.0022 (6)
C8	0.0181 (7)	0.0273 (9)	0.0288 (8)	0.0025 (7)	0.0060 (7)	−0.0032 (7)
C9	0.0217 (8)	0.0232 (9)	0.0234 (8)	0.0040 (7)	0.0018 (6)	0.0020 (6)
C10	0.0230 (8)	0.0205 (8)	0.0183 (7)	0.0006 (6)	0.0060 (6)	0.0010 (6)
C11	0.0225 (7)	0.0137 (8)	0.0169 (7)	−0.0013 (6)	0.0056 (6)	−0.0003 (6)
C12	0.0205 (7)	0.0178 (8)	0.0145 (7)	0.0001 (6)	0.0051 (6)	−0.0005 (6)
C13	0.0204 (7)	0.0142 (8)	0.0178 (7)	−0.0013 (6)	0.0040 (6)	−0.0009 (6)
C14	0.0210 (7)	0.0231 (9)	0.0202 (7)	0.0018 (6)	0.0072 (6)	−0.0007 (6)
C15	0.0168 (7)	0.0225 (9)	0.0149 (7)	0.0027 (6)	0.0046 (6)	0.0013 (6)
C16	0.0205 (7)	0.0180 (8)	0.0188 (7)	0.0039 (6)	0.0039 (6)	−0.0011 (6)
C17	0.0189 (7)	0.0174 (8)	0.0205 (7)	0.0011 (6)	0.0045 (6)	0.0034 (6)
C18	0.0151 (7)	0.0215 (8)	0.0145 (7)	0.0041 (6)	0.0030 (6)	0.0041 (6)
C19	0.0210 (7)	0.0194 (8)	0.0170 (7)	0.0009 (6)	0.0039 (6)	−0.0022 (6)
C20	0.0202 (7)	0.0197 (8)	0.0191 (7)	−0.0020 (6)	0.0056 (6)	0.0001 (6)
C21	0.0235 (8)	0.0273 (9)	0.0178 (7)	0.0040 (7)	0.0083 (6)	−0.0011 (6)

### Geometric parameters (Å, °)

**Table:**

O1—C1	1.3259 (17)	C8—C9	1.380 (2)
O1—H1	0.864 (10)	C8—H8	0.9500
O2—C11	1.2950 (17)	C9—C10	1.390 (2)
O3—C18	1.3657 (17)	C9—H9	0.9500
O3—C21	1.4291 (19)	C10—H10	0.9500
N1—C1	1.3484 (19)	C11—C12	1.410 (2)
N1—N2	1.3981 (16)	C12—C13	1.381 (2)
N1—C5	1.4208 (19)	C12—H12	0.9500
N2—C3	1.3213 (19)	C13—C14	1.499 (2)
N3—C13	1.3410 (19)	C14—H14A	0.9800
N3—C15	1.4350 (19)	C14—H14B	0.9800
N3—H3	0.888 (9)	C14—H14C	0.9800
C1—C2	1.390 (2)	C15—C20	1.383 (2)
C2—C3	1.4277 (19)	C15—C16	1.389 (2)
C2—C11	1.445 (2)	C16—C17	1.384 (2)
C3—C4	1.490 (2)	C16—H16	0.9500
C4—H4A	0.9800	C17—C18	1.392 (2)
C4—H4B	0.9800	C17—H17	0.9500
C4—H4C	0.9800	C18—C19	1.391 (2)
C5—C10	1.387 (2)	C19—C20	1.393 (2)
C5—C6	1.391 (2)	C19—H19	0.9500
C6—C7	1.383 (2)	C20—H20	0.9500
C6—H6	0.9500	C21—H21A	0.9800
C7—C8	1.386 (2)	C21—H21B	0.9800
C7—H7	0.9500	C21—H21C	0.9800
			
C1—O1—H1	99.0 (16)	C9—C10—H10	120.3
C18—O3—C21	117.30 (12)	O2—C11—C12	121.30 (13)
C1—N1—N2	110.07 (12)	O2—C11—C2	115.32 (13)
C1—N1—C5	130.27 (12)	C12—C11—C2	123.38 (13)
N2—N1—C5	119.65 (11)	C13—C12—C11	124.10 (13)
C3—N2—N1	105.77 (11)	C13—C12—H12	118.0
C13—N3—C15	125.88 (13)	C11—C12—H12	118.0
C13—N3—H3	114.1 (12)	N3—C13—C12	122.10 (13)
C15—N3—H3	119.1 (12)	N3—C13—C14	117.52 (13)
O1—C1—N1	124.40 (13)	C12—C13—C14	120.35 (13)
O1—C1—C2	126.92 (14)	C13—C14—H14A	109.5
N1—C1—C2	108.68 (12)	C13—C14—H14B	109.5
C1—C2—C3	103.98 (13)	H14A—C14—H14B	109.5
C1—C2—C11	119.59 (13)	C13—C14—H14C	109.5
C3—C2—C11	136.42 (14)	H14A—C14—H14C	109.5
N2—C3—C2	111.50 (13)	H14B—C14—H14C	109.5
N2—C3—C4	119.49 (12)	C20—C15—C16	119.82 (14)
C2—C3—C4	129.00 (13)	C20—C15—N3	120.37 (14)
C3—C4—H4A	109.5	C16—C15—N3	119.71 (14)
C3—C4—H4B	109.5	C17—C16—C15	120.12 (15)
H4A—C4—H4B	109.5	C17—C16—H16	119.9
C3—C4—H4C	109.5	C15—C16—H16	119.9
H4A—C4—H4C	109.5	C16—C17—C18	120.05 (15)
H4B—C4—H4C	109.5	C16—C17—H17	120.0
C10—C5—C6	120.45 (14)	C18—C17—H17	120.0
C10—C5—N1	120.55 (13)	O3—C18—C19	124.54 (14)
C6—C5—N1	119.00 (13)	O3—C18—C17	115.33 (14)
C7—C6—C5	119.44 (14)	C19—C18—C17	120.13 (14)
C7—C6—H6	120.3	C18—C19—C20	119.28 (14)
C5—C6—H6	120.3	C18—C19—H19	120.4
C6—C7—C8	120.57 (14)	C20—C19—H19	120.4
C6—C7—H7	119.7	C15—C20—C19	120.60 (15)
C8—C7—H7	119.7	C15—C20—H20	119.7
C9—C8—C7	119.60 (15)	C19—C20—H20	119.7
C9—C8—H8	120.2	O3—C21—H21A	109.5
C7—C8—H8	120.2	O3—C21—H21B	109.5
C8—C9—C10	120.63 (15)	H21A—C21—H21B	109.5
C8—C9—H9	119.7	O3—C21—H21C	109.5
C10—C9—H9	119.7	H21A—C21—H21C	109.5
C5—C10—C9	119.30 (14)	H21B—C21—H21C	109.5
C5—C10—H10	120.3		
			
C1—N1—N2—C3	0.61 (16)	N1—C5—C10—C9	179.96 (14)
C5—N1—N2—C3	−177.93 (13)	C8—C9—C10—C5	0.2 (2)
N2—N1—C1—O1	178.89 (14)	C1—C2—C11—O2	−3.6 (2)
C5—N1—C1—O1	−2.8 (3)	C3—C2—C11—O2	176.96 (17)
N2—N1—C1—C2	−0.54 (17)	C1—C2—C11—C12	175.40 (15)
C5—N1—C1—C2	177.79 (15)	C3—C2—C11—C12	−4.1 (3)
O1—C1—C2—C3	−179.15 (15)	O2—C11—C12—C13	7.2 (2)
N1—C1—C2—C3	0.26 (17)	C2—C11—C12—C13	−171.74 (15)
O1—C1—C2—C11	1.2 (2)	C15—N3—C13—C12	169.33 (15)
N1—C1—C2—C11	−179.38 (13)	C15—N3—C13—C14	−12.5 (2)
N1—N2—C3—C2	−0.44 (17)	C11—C12—C13—N3	−1.7 (2)
N1—N2—C3—C4	−179.84 (13)	C11—C12—C13—C14	−179.77 (15)
C1—C2—C3—N2	0.12 (18)	C13—N3—C15—C20	109.16 (18)
C11—C2—C3—N2	179.67 (17)	C13—N3—C15—C16	−74.5 (2)
C1—C2—C3—C4	179.46 (15)	C20—C15—C16—C17	0.7 (2)
C11—C2—C3—C4	−1.0 (3)	N3—C15—C16—C17	−175.75 (13)
C1—N1—C5—C10	−20.5 (2)	C15—C16—C17—C18	−0.3 (2)
N2—N1—C5—C10	157.74 (14)	C21—O3—C18—C19	0.5 (2)
C1—N1—C5—C6	159.97 (15)	C21—O3—C18—C17	−179.53 (13)
N2—N1—C5—C6	−21.8 (2)	C16—C17—C18—O3	179.91 (13)
C10—C5—C6—C7	0.3 (2)	C16—C17—C18—C19	−0.2 (2)
N1—C5—C6—C7	179.88 (14)	O3—C18—C19—C20	−179.88 (13)
C5—C6—C7—C8	0.2 (2)	C17—C18—C19—C20	0.2 (2)
C6—C7—C8—C9	−0.5 (3)	C16—C15—C20—C19	−0.6 (2)
C7—C8—C9—C10	0.3 (3)	N3—C15—C20—C19	175.76 (13)
C6—C5—C10—C9	−0.5 (2)	C18—C19—C20—C15	0.2 (2)

### Hydrogen-bond geometry (Å, °)

**Table:**

Cg1 and Cg2 are the centroids of the N1,N2,C1–C3 and C15–C20 rings, respectively.

**Table:**

*D*—H···*A*	*D*—H	H···*A*	*D*···*A*	*D*—H···*A*
O1—H1···O2	0.86 (1)	1.68 (1)	2.4963 (15)	156 (2)
N3—H3···O2	0.89 (1)	1.92 (1)	2.6447 (16)	138.(2)
C14—H14b···Cg1^i^	0.98	2.88	3.5542 (18)	127
C21—H21c···Cg2^ii^	0.98	2.76	3.5195 (17)	134

Symmetry codes: (i) −*x*+1, −*y*+1, −*z*+1; (ii) −*x*+2, −*y*+1, −*z*.
